# αβT/CD19-depleted Allogeneic Stem Cell Transplantation in Adults with Inborn Errors of Immunity

**DOI:** 10.1007/s10875-025-01978-9

**Published:** 2026-02-03

**Authors:** Janneke J. H. de Winter, Birtan M. Ibrahimov, Frances A. Verheij, Iris D. Brinkman, Anniek H. G. Stuut, Pleun Schonewille, Marloes W. Heijstek, Anna van Rhenen, Lotte E. van der Wagen, Laura G. M. Daenen, Anke Janssen, Tim J. A. Hutten, Jürgen Kuball, Helen L. Leavis, Moniek A. de Witte

**Affiliations:** 1 Department of Hematology, University Medical Center Utrecht, Heidelberglaan 100, 3584 CX Utrecht, The Netherlands; 2 Department of Hematology, Isala Clinics, Zwolle, Netherlands; 3 Department of Hematology and Center of Translational Immunology, University Medical Center Utrecht, Utrecht, Netherlands; 4 Department of Rheumatology and Clinical Immunology, University Medical Center Utrecht, Utrecht, Netherlands; 5 Central Diagnostic Laboratory, University Medical Center Utrecht, Utrecht, Netherlands

**Keywords:** Inborn errors of immunity (IEI), Allogeneic hematopoietic stem cell transplantation (allo-HSCT), Graft engineering, Graft-versus-host disease (GvHD), Autoimmunity, Immunodeficiency

## Abstract

**Purpose:**

Allogeneic hematopoietic stem cell transplantation (allo-HSCT) is successful in pediatric patients with inborn errors of immunity (IEI), but its use in adults is complicated by pre-existing organ damage and increased risk of treatment-related mortality. *Ex vivo* graft engineering using αβTCR/CD19 depletion has shown promising safety profiles in pediatric IEI, yet evidence in adults is limited. We assessed the feasibility and outcomes of αβTCR/CD19-depleted allo-HSCT in adults with IEI, focusing on engraftment, immune reconstitution, and clinical outcomes.

**Methods:**

We included 9 adults with IEI and 1 with VEXAS (age 21–51). IEIs included CTLA4HI, APDS, DOCK8, ALPS, DADA2, CVID2, and HA20, with Immune Deficiency and Dysregulation Activity (IDDA) scores of 17–92. αβTCR/CD19-depleted allografts from related, unrelated or haplo-identical donors were used after antithymocyte globulin (ATG) and myeloablative conditioning (thiotepa, melphalan, and fludarabine). Post-transplant immunoprophylaxis included mycophenolate mofetil; 4/10 patients received additional transplant-associated immunosuppression.

**Results:**

All patients achieved primary engraftment. One patient with secondary rejection successfully underwent a second allo-HSCT. 5 patients developed grade 2–4 acute GvHD; no chronic GvHD was observed. One patient with GvHD died from COVID-19. All remaining 9 patients were successfully tapered off immunosuppression and showed improved IDDA scores. At 6 months NK, γδT, B and CD8 + T cells normalized; CD4 + numbers reached 149 cells/µl at 1 year. Most patients were successfully vaccinated and could stop immunoglobulin substitution.

**Conclusion:**

In conclusion, *ex vivo* graft engineering using αβTCR/CD19 depletion was feasible in adults with IEI. Clinical outcomes are encouraging, but need to be confirmed in larger studies.

**Supplementary Information:**

The online version contains supplementary material available at 10.1007/s10875-025-01978-9.

## Introduction

Inborn errors of immunity (IEI) comprise a heterogeneous group of diseases caused by genetic defects that impair the immune system’s ability to mount an appropriate response. Patients with IEI often develop severe infections and immune dysregulation, including autoimmunity, autoinflammation, and an increased risk of malignancy. In children with severe IEI, allogeneic hematopoietic stem cell transplantation (allo-HSCT) is the standard of care and offers a high cure rate, particularly when performed before the onset of organ damage or infectious complications [1–4]. In adults, allo-HSCT shows promise for selected patients. A large retrospective multicenter study on allo-HSCT outcomes, including 329 patients with IEI, reported an estimated 1-year overall survival (OS) of 78% and an event-free survival (EFS) of 65% [5]. While long-term outcomes appear to favor allo-HSCT, its early toxicity presents significant challenges. These include 13% grade III-IV acute graft-versus-host disease (aGvHD), 4% extensive chronic GvHD (cGvHD), 14% graft failure, 18% bacterial infections, and 25% viral reactivations, resulting in a 1-year transplant-related mortality (TRM) of 13%. These hurdles must be addressed before allo-HSCT can be routinely offered to a broader adult patient population with IEI.

Since αβ T cells and B cells play a key role in the pathophysiology of GvHD [6, 7], the combination of αβ TCR and CD19 depletion has been successfully introduced in haploidentical allo-HSCT for children with non-malignant disorders [8]. This approach has since demonstrated promising outcomes across all donor types in both children and adults with hematological malignancies, with very low rates of cGvHD [9–12]. A recent European Society for Blood and Marrow Transplantation (EBMT) survey analyzing 167 children with IEI who received ex vivo αβTCR/CD19-depleted allo-HSCT reported an OS of 78% and a low rate of chronic GvHD of 7% [13].

Based on a recent study where we show favorable engraftment rates and low incidence of severe GVHD after transplantation with αβTCR/CD19 depleted 10/10 or 9/10 matched allografts in adult patients with malignant diseases [11], we considered this approach especially attractive in patients with non-malignant diseases such as IEI. Here, we report the first 10 consecutive adult patients with IEI who underwent αβTCR/CD19-depleted allo-HSCT.

## Methods

This retrospective analysis included all adult patients with inborn errors of immunity (IEI), including one patient with VEXAS syndrome (associated with a somatic mutation in the UBA1 gene), who received an αβTCR/CD19-depleted allo-HSCT between 2019 and 2024 at the University Medical Center Utrecht (UMCU), The Netherlands. All patients provided written informed consent indicating their agreement to participate in the Dutch National ‘IEI’ registry and gave consent which allowed their data to be sent to EBMT. The informed consent form was approved by the local ethical committee (METC NL40331.078 & METC nr 21–322) at the UMCU. The study adhered to Good Clinical Practice (GCP) protocols and the principles outlined in the Declaration of Helsinki. Eligibility criteria included age ≥ 18 years at the time of allo-HSCT, a confirmed clinical and/or genetic diagnosis, and a first allo-HSCT performed between 2019 and 2023. Clinical trial number: not applicable.

Basic demographic data included age, sex, IEI diagnosis and/or genetic diagnosis, age at allo-HSCT, and HCT comorbidity index [14]. Transplant-specific demographics included donor type, cell source, conditioning regimen, and donor/patient cytomegalovirus (CMV) and Epstein-Barr virus (EBV) serostatus. The indication for allo-HSCT was determined based on international guidelines [12, 15], with key clinical features serving as the primary criteria for allo-HSCT. Table 1 shows baseline characteristics of the included patients.

**Table 1:** Baseline characteristics

Patient Information							Immune therapy	Patient scores			
#	Sex	Age	Diagnosis	CMV status (Patient/Donor)	EBV status (Patient/Donor)	Donor type	Immune therapy at SCT	IDDA baseline	Karnofsky score	HCT-CI	Key clinical features
1	F	34	CTLA4HI	Pos/ Neg	Pos/ Pos	Haploidentical donor	MMF + Rituximab	54	70	2	GLILD, CVID
2	M	40	APDS	Pos/ Pos	Pos/ ?	MUD 9/10	None	62	60	4	PTLD
3	M	24	DOCK8	Neg/ Neg	Pos/ Neg	MUD 10/10	None	17	90	0	Refractory eczema, infections
4	M	30	ALPS	Pos/ Pos	Pos/ Pos	MUD 10/10	None	26	80	0	Recurrent Hodgkin’s lymphoma
5	M	30	DADA2	Pos/ Pos	Pos/ Pos	MRD 10/10	Infliximab + CsA	54	70	1	DLBCL
6	M	40	CTLA4HI	Neg/ Neg	Neg/ Neg	MUD 10/10	Prednisolone + Abatacept	62	60	3	GLILD
7	M	51	VEXAS syndrome	Pos/ Pos	Neg/ Pos	MUD 9/10	Prednisolone + Canakinumab	39	80	2	Cytopenia, auto-inflammation
8	F	28	CVID2 (TACI)	Pos/ Pos	Pos/ Pos	MUD 10/10	None	34	80	0	Hodgkin’s lymphoma
9	F	23	IEI HA20	Pos/ Pos	Pos/ Pos	MUD 9/10	Prednisolone + Ruxolitinib + Anakinra	92	40	1	Refractory colitis, auto-inflammation
10	M	21	DADA2	Pos/ Pos	Pos/ ?	MUD 10/10	Prednisolone + Golimumab	22	80	6	Hepatobiliary inflammation, vasculitis

*ALPS* Autoimmune Lymphoproliferative Syndrome; *APDS* Activated PI3K Delta Syndrome; *CMV* Cytomegalovirus; *CsA* Cyclosporine A; *CTLA4HI* Cytotoxic T−lymphocyte Associated Protein 4 Haploinsufficiency; *CVID* Common Variable Immunodeficiency; *DADA2* Adenosine Deaminase 2 Deficiency; *DLBCL* Diffuse Large B−Cell Lymphoma; *DOCK* 8 Dedicator of Cytokinesis 8 Deficiency; *EBV* Epstein–Barr Virus; *GLILD* Granulomatous−lymphocytic Interstitial Lung Disease; *HCT−CI* Hematopoietic Cell Transplantation−specific Comorbidity Index; *IDDA* Immune Deficiency and Dysregulation Activity; *IEI* Inborn Errors of Immunity; *MDS* Myelodysplastic Syndromes; *MMF* Mycophenolate Mofetil; MRD Matched Related Donor; *MUD* Matched Unrelated Donor; *PTLD* Post−transplant Lymphoproliferative Disorder; *TACI* Transmembrane Activator and CAML Interactor mutation; *VEXAS* Syndrome Vacuoles, E1 Enzyme, X−linked, Autoinflammatory, Somatic Syndrome

Myeloablative conditioning consisted of anti-thymocyte globulin (ATG)-Fresenius, fludarabine, thiotepa and melphalan, as previously described [12]. One patient underwent a second allo-HSCT after experiencing secondary graft failure, using a conditioning regimen of ATG, fludarabine and treosulfan followed by a T cell replete allo-HSCT. T and B cells were depleted ex vivo by anti-αβ T cell receptor (TCR) and CD19 antibodies [12]. Mycophenolate mofetil (MMF) was administered at a dose of 15 mg/kg, three times daily (maximum 3000 mg) for 28 days as the sole prophylaxis for GvHD. In patients requiring transplant-associated immunosuppression prior to allo-HSCT a personalized tapering scheme after stem cell infusion was designed (Table 1). Short tandem repeat (STR) method was used for the calculation of chimerism [16]. Mixed chimerism was defined as < 95% donor chimerism in the non-T-cell fraction [17]. A pre-emptive donor lymphocyte infusion (DLI) was administered in case of decline in donor chimerism of infectious complications in the absence of active GvHD.

Neutrophil recovery was defined as the first of 3 consecutive days with an absolute neutrophil count > 0.5 × 10^9^/L. Secondary graft failure was defined as the development of pancytopenia after initial engraftment, with a loss of donor chimerism beyond day 28. aGvHD and cGvHD were graded according to Glucksberg [18]. Graft and Relapse-free survival (GRFS) was defined according to the definition of Ruggeri et al. [19]. Overall survival (OS) was defined as time from allo-HSCT to death from any cause, censoring at the final day of follow-up. Event-free survival (EFS) was defined as time from transplantation to relapse, graft failure or death, censoring at the last day of follow-up. CMV reactivation was defined as a viral load of > 250 IU/ml, EBV reactivation was defined as > 1000 IU/ml as assessed by polymerase chain reaction (PCR). Letermovir prophylaxis was implemented in our institute as CMV prophylaxis since December 2022 in CMV seropositive recipients. Patient #9 and #10 received letermovir prophylaxis until day 100 post allo-SCT.

Patients with less than 2 years of follow-up were censored at their last follow-up date. The Immune Deficiency and Dysregulation Activity (IDDA) score provides a standardized tool to track disease morbidity and clinical symptoms over time [20]. The IDDA scores were calculated at the time of allo-HSCT indication, 1 year after allo-HSCT and at the latest follow-up. Pneumococcal seroconversion was categorized as follows: "not protected" if fewer than six serotypes exhibited antibody concentrations ≤ 0.35 µg/mL, "well protected" if six or more serotypes exhibited antibody concentrations ≥ 1.00 µg/mL, and "moderately protected" if neither of these criteria was fulfilled.

Patient and transplant characteristics were expressed as the number and percentage of the group for categorical variables and median with ranges for continuous variables. Kaplan–Meier curves of overall OS, EFS and GRFS were plotted. Relapse, non-relapse mortality (NRM) and aGvHD were calculated as cumulative incidence (CI) curves to adjust the estimates for competing risks. R version 4.3.3 was used for statistical analysis and creating figures.

## Results

### Patient and Transplantation Characteristics

Ten patients with a variety of IEI were included in this study; haploinsufficiency of CTLA4 (CTLA4HI) (*n* = 2), activated PI3Kdelta syndrome (APDS) (*n* = 1), DOCK 8 deficiency (*n* = 1), ALPS (*n* = 1), ADA2 deficiency (DADA2) (*n* = 2), VEXAS (vacuoles, E1 enzyme, X-linked, autoinflammatory, somatic) syndrome (*n* = 1), CVID type 2 (mutation in transmembrane activator and CAML interactor (TACI)) (*n* = 1) and haploinsufficiency of A20 (HA20) (*n* = 1) (Table 1; details on mutations in Online Resource Table S1). Three of the 10 patients were female (30%). Mean age at allo-HSCT was 32.1 (range 21–51). Eight out of 10 patients (80%) were at risk for CMV reactivation based on CMV serostatus combinations (patient/donor: Pos/Pos, Pos/Neg). One patient was transplanted with a 10/10 matched related donor, five of the donors were 10/10 matched unrelated (50%) and four patients received mismatched transplants (9/10 matched unrelated (*n* = 3) and haplo-identical (*n* = 1)). The median baseline IDDA score was 46 (range 17–92), with a median Karnofsky score of 75 (range 40–90) and median HCT comorbidity index score of 1.5 (range 0–6). The primary indication for allo-HSCT was malignancy in four patients (40%), auto-inflammatory disease in five patients (50%), and infection in one patient (10%). Six patients were dependent on systemic immunosuppression prior to allo-HSCT to control autoimmunity.

### Graft-failure, Graft-Versus-Host Disease and Infections

**Fig. 1:**
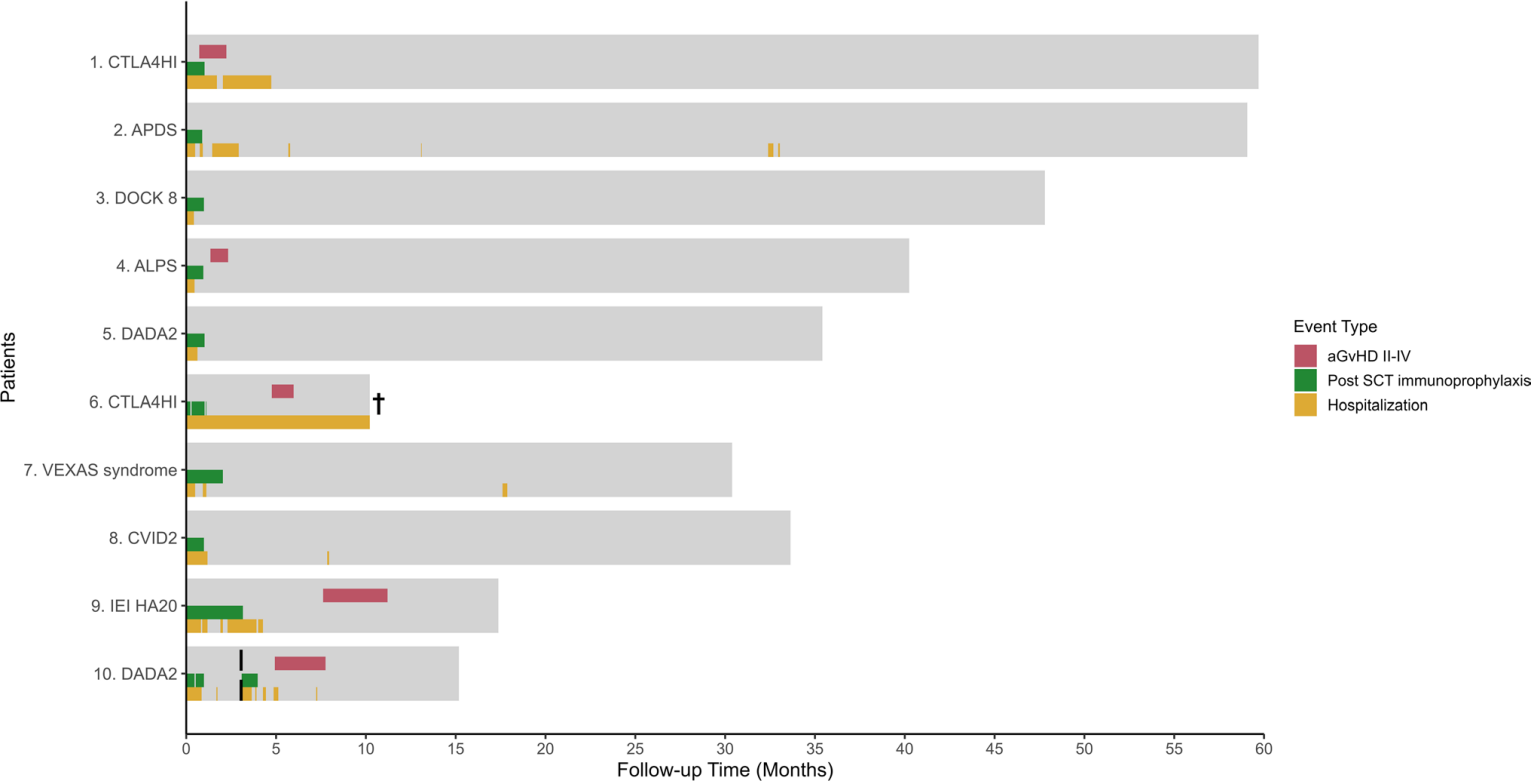
Follow-up time with events and hospitalizations after stem cell transplantation. Follow-up times for ten patients with inborn errors of immunity, highlighting acute graft-versus-host disease (aGvHD) grades II-IV (upper band), personalized post-SCT (stem cell transplantation) immunoprophylaxis (middle band), hospitalizatons (lower band), and one second stem cell transplantation (dashed vertical line) due to graft rejection. † Indicates one death

The depletion procedure was successful in all cases. The median number of infused CD34 + cells was 5.93 × 10^6^/kg (range 3.42 to 7.47) and the median number of infused αβTCR cells and B cells were 0.009 × 10^6^/kg (range 0.001 to 0.199) and 0.019 × 10^6^/kg (range 0.007 to 0.042) respectively (Online Resource Table S2). All patients engrafted with a median of 13 days. One patient (patient #10) developed a secondary graft-failure, potentially induced by a human herpesvirus 6 (HHV-6) infection (Table 1, Fig. 1). This patient successfully received a second transplantation with a T cell replete allo-HSCT (Fig. 1). Four patients developed aGvHD of grade 2–4, of which two patients had a grade 3–4 aGvHD. Grade 3–4 aGvHD occurred in patient #1 with CTLA4HI and patient #10 with DADA2 after the second T cell replete allo-HSCT. In all patients, immunosuppression medication could be successfully tapered off. No cases of limited or extensive chronic GvHD were reported.

Based on the two cases with CTLA4HI who developed aGvHD, we hypothesized that for patients requiring systemic disease-associated immunosuppression prior to allo-HSCT, single agent post-transplant immune prophylaxis may be insufficient to prevent GvHD development even after stringent αβTCR/CD19 depletion. Hence, we started tapering the disease-associated immunosuppression only after the MMF could successfully be stopped at day 28 in patient #7, #9 and #10 (Table 2 and Fig. 1). The incidence of CMV reactivation in CMV-seropositive patients was 50%. No patient experienced EBV reactivation after αβTCR/CD19 depletion, although patient #10 did develop EBV reactivation after the second SCT, which was likely transferred by the donor B cells.

**Table 2:** Outcomes after stem cell transplantation

Patient Information			Immune Therapy	IDDA scores				Donor chimerism*	
#	Diagnosis	Days to engraftment (Median: 13)	After SCT	Baseline	1 Year after SCT	At Latest Follow-up	Follow-up time (months)	T cell	Non-T cell
1	CTLA4HI	10	MMF	54	20	6	59	100	100
2	APDS	23	MMF	62	0	0	59	100	100
3	DOCK8	16	MMF	17	2	0	47	96	99
4	ALPS	14	MMF	26	0	0	40	100	100
5	DADA2	36	MMF	54	10	2	35	78	97
6	CTLA4HI †	9	MMF + Prednisolone + Abatacept	62	199	-	10	98	98
7	VEXAS syndrome	0	MMF + Prednisolone + Canikunumab	39	4	2	30	100	100
8	CVID2 (TACI)	14	MMF	34	7	4	33	94	96
9	IEI HA20	11	MMF + Prednisolone + Ruxolitinib + Anakinra	92	24	-	17	100	100
10	DADA2	12	MMF + Prednisolone + Hydrocortisone	22	0	-	15	5/100**	15/100**

^†^Indicates death; *The last measured chimerism value; **Values from the first/second hematopoietic stem cell transplantation, respectively

*ALPS* Autoimmune Lymphoproliferative Syndrome; *APDS* Activated PI3K Delta Syndrome; *CTLA4HI* Cytotoxic T−lymphocyte Associated Protein 4 Haploinsufficiency; *CVID* Common Variable Immunodeficiency; *DADA2* Adenosine Deaminase 2 Deficiency; *DOCK 8* Dedicator of Cytokinesis 8 Deficiency; *IDDA* Immune Deficiency and Dysregulation Activity; *IEI* Inborn Errors of Immunity; *MMF* Mycophenolate Mofetil; *TACI* Transmembrane Activator and CAML Interactor mutation; *VEXAS syndrome* Vacuoles, E1 Enzyme, X−linked, Autoinflammatory, Somatic Syndrome

Regarding infectious complications, patient #6 died of COVID 19 infection, patient #10 developed an HHV-6 infection that precipitated secondary rejection and patient #2 had adenoviremia, which was successfully treated with cidofovir and DLI (see below). Four out of 10 patients were readmitted within the first year for: GvHD, ileostomy removal, graft failure or kidney disfunction. Ultimately, these complications resulted in a NRM rate of 13% (n = 1) at 2 years (data not shown).

### Immune Reconstitution

**Fig. 2:**
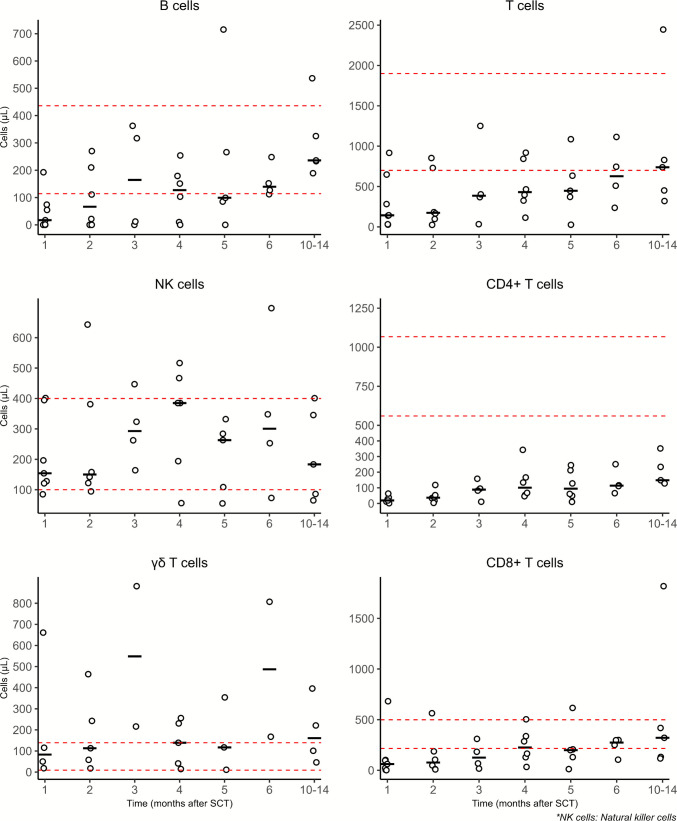
Immune reconstitution after hematopoietic stem cell transplant, showing the amounts of B cells, T cells, NK cells, CD4 + T cells, γδ T cells, and CD8 + T cells at different time points. Immune cells were measured at 1, 2, 3, 4, 5, 6, and 10–14 months post-stem cell transplantation. Cell counts are per µL, with red dashed lines indicating reference ranges. Black horizontal lines represent the median. Circles represent mean measurements for that time period for individual patients. Patient #10 was excluded, as he received a second T cell replete allo-HSCT. NK cells: natural killer cells

Immune reconstitution of B cells, T cells, NK cells, CD8 + T cells, CD4 + T cells, and γδT cells was measured at standard timepoints (Fig. 2). NK cells (median of 154 cells/μL (range 85–402)) and γδT (median of 83 cells/μL cells (range 18–661)) normalized within 1 month after transplantationInterestingly, B cells (median 140 cells/μL, range 112–248) and CD8 + T cells (median 274 cells/μL, range 105–299) also normalized within 6 months in most patients. As expected, CD4 + T cells take longer to normalize and reach a median of 149 (range 128–352) cells/μL at 10–14-months.

**Fig. 3:**
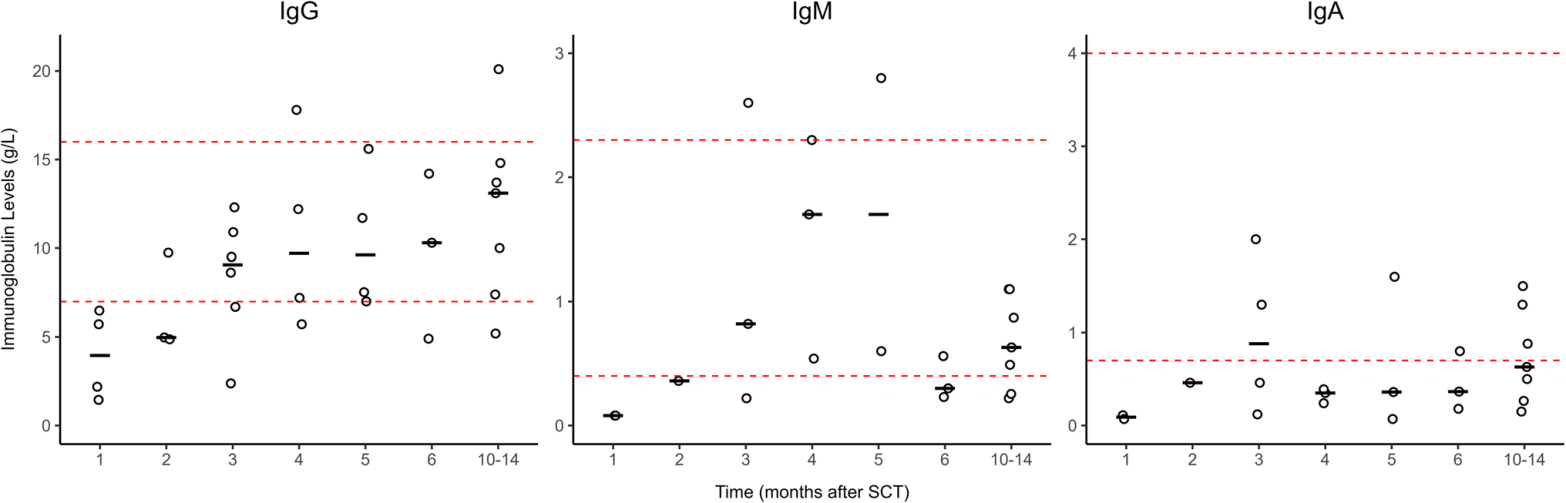
Immunoglobulin levels at different time points after transplantation. Immunoglobulin (Ig) levels measured at 1, 2, 3, 4, 5, 6, and 10–14 months post-stem cell transplantation, shown in g/L. Red dashed lines indicate reference ranges. Each black horizontal line represents the median. Each circle is the mean measurement for that time period for each individual patient

B-cell recovery leads to normalization of immunoglobulins, as shown in Fig. 3. At 3 months after transplantation, median IgG levels were 8.6 (range 2.4–12.3) g/L, median IgM 0.8 (range 0.2–2.6) g/L, and median IgA 0.9 (range 0.12–2) g/L. Nine patients (90%) received immunoglobulin replacement therapy prior to allo-HSCT. At the most recent follow-up, only one patient (#8) still required immunoglobulin replacement therapy. Vaccination response titers against *Streptococcus pneumoniae* were measured in seven out of the 10 patients (70%). Patient #6 died before vaccination administration, and patients #9 and #10 had not yet completed their vaccination regimen at the time of data cut-off. Only one patient (14.3%) was classified as not protected, while five (71.4%) were moderately protected, and 1 (14.3%) was well protected (Online Resource Fig. S1).

### Chimerism and Donor Lymphocyte Infusions

**Fig. 4:**
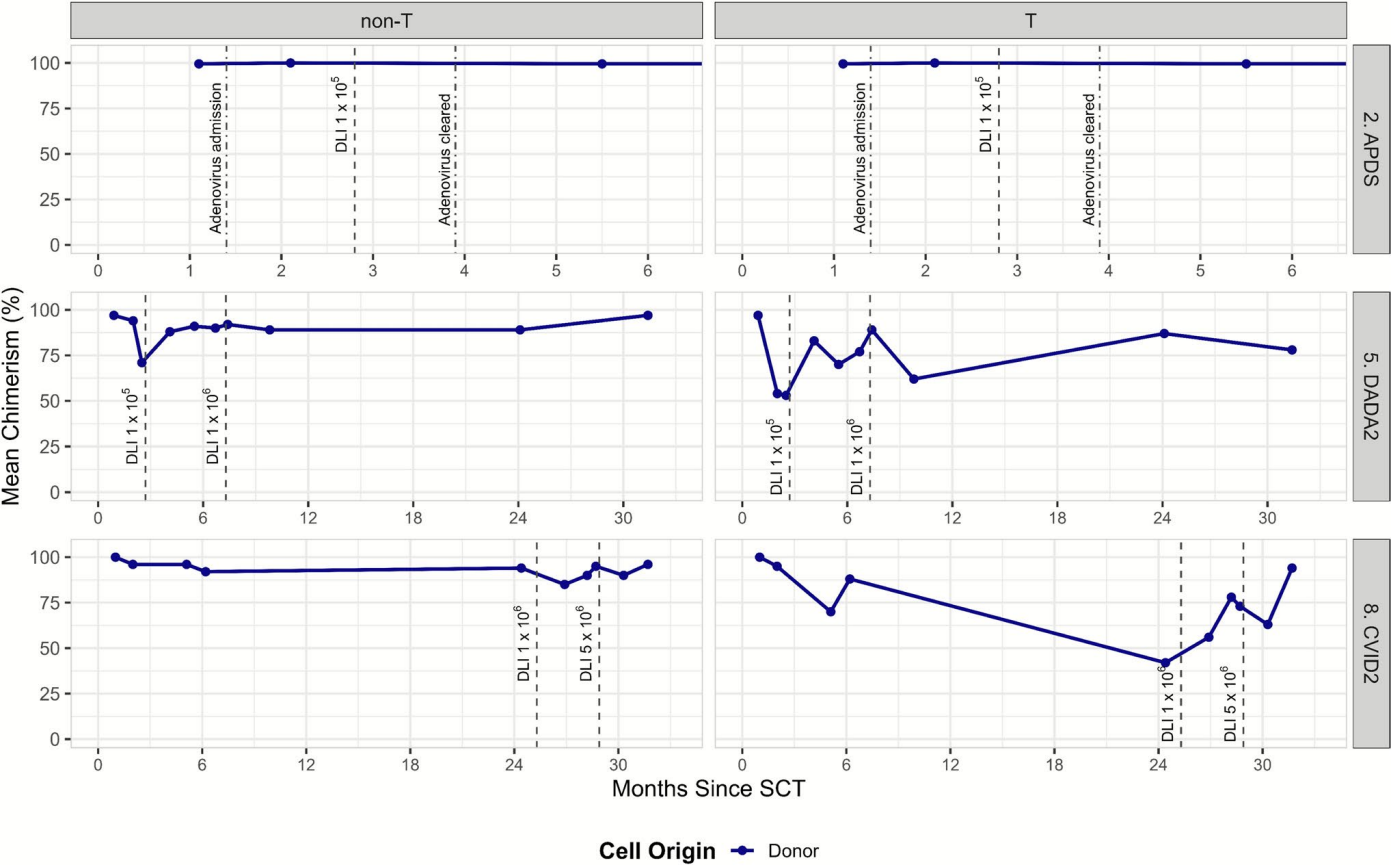
Donor chimerism over time in T and non-T cell populations post-transplant. Time course of donor chimerism in T and non-T cells for three patients (#2. APDS, #5. DADA2, #8. CVID2) post-stem cell transplantation. Mean chimerism percentages are shown, with key clinical events (adenovirus infection and donor lymphocyte infusions in cells/kg) marked by dot-dashed and dashed lines, respectively

Chimerism data were available for all patients at the most recent follow-up. For patient #10, the reported chimerism values reflect the second allo-HSCT, following the secondary graft failure of the first transplant (Table 2). The median donor chimerism of T-cell was 100% (range 78–100%), and the median donor chimerism of non-T cells was 100% (range 96–100%). At the latest follow-up, all surviving patients demonstrated stable full donor chimerism. Patients #5 and #8 showed mixed chimerism (Fig. 4). Both patients achieved full chimerism after pre-emptive DLI. In patient #3 and #6 a prophylactic DLI (pro-DLI) of 1 × 10^5^ T cells/kg were administered as standard of care to restore immune reconstitution. No GvHD was observed after these pro-DLIs. Patient #1 and #4 did not receive a pro-DLI due to active GvHD, patient #7 and #9 due to active transplant-associated immunosuppression, and patient #10 due to the rapid decline in donor chimerism level and need for a second transplant.

Patient #2 developed adenoviremia. As the viral load did not respond to anti-viral medication, a pre-emptive DLI of 1 × 10^5^ T cells/kg was given at 85 days after transplant. After 34 days the adenovirus load was undetectable in the peripheral blood and the patient did not develop GvHD (Fig. 4). Patient #5 and patient #8 received a pre-emptive DLI because of a decline in donor chimerism. In both cases donor chimerism improved after 2 DLIs with increasing dose (Fig. 4).

### Clinical Outcome

The median follow-up to assess long-term outcome of this cohort was 3 years (range: 1–5 years) (Table 2, Fig. 1). Disease activity was assessed by IDDA scores [20]. Among the nine patients who were alive, the IDDA score improved in all 9 patients, with a median IDDA score of 4 (range 0–24) one year after allo-HSCT (Table 2, Online Resource Fig. S2). At final follow-up, the median IDDA score was 2 (range 0–6). The median decrease in IDDA score was 3 (range 15–68) at one-year follow-up, and 37 (range 17–62) at latest follow-up. Patient #6 with CTLA4 haploinsufficiency (CTLA4-HI), who died from a COVID-19 infection, had a high final IDDA score of 199.

**Fig. 5:**
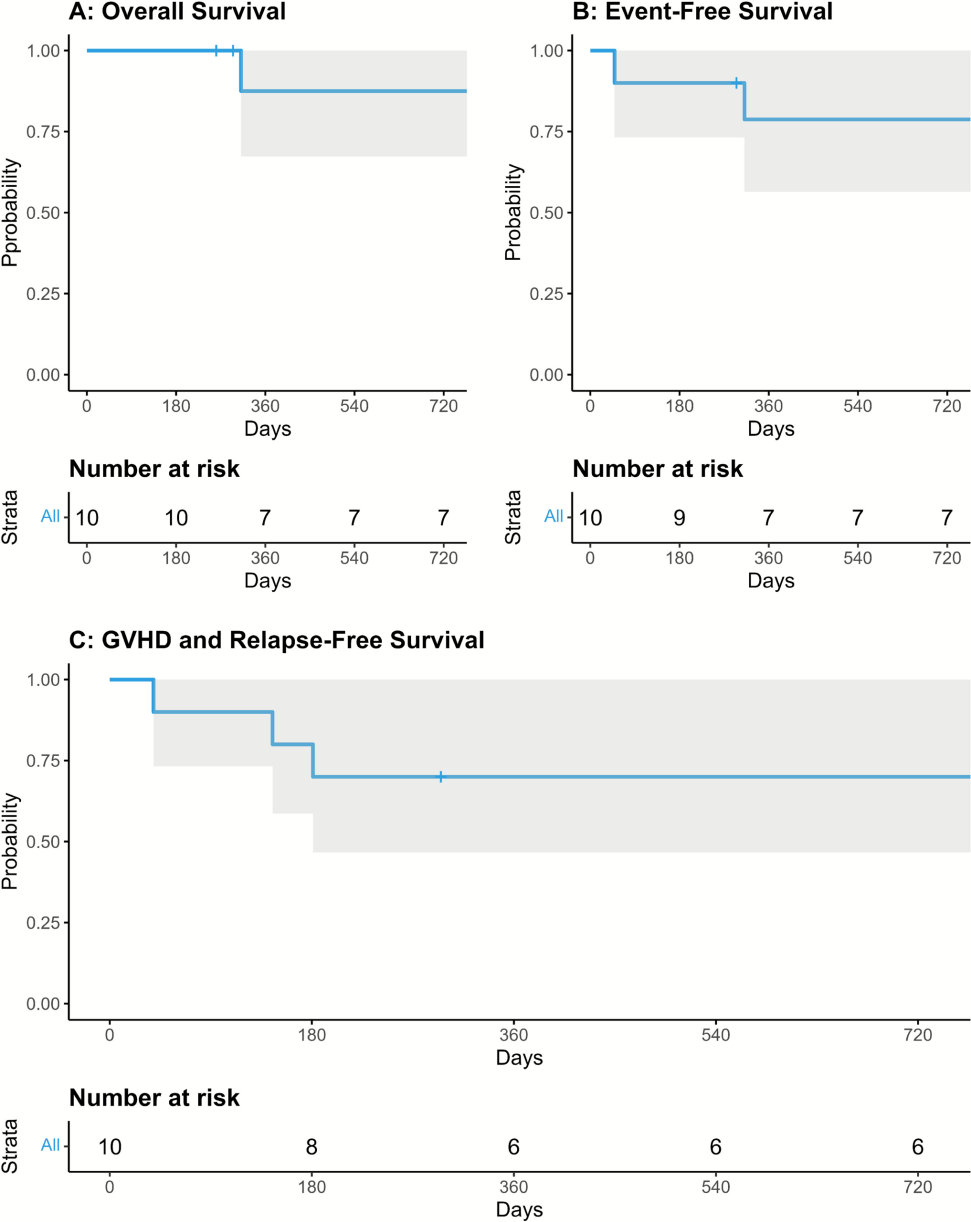
Post-transplantation outcomes: survival and mortality. Kaplan–Meier plots for Overall Survival (**A**), Event-Free Survival (**B**), Graft-versus-Host Disease-Free and Relapse-Free Survival (**C**) over 2 years post-transplantation. Shaded areas indicate 95% confidence intervals, and “Number at risk” tables detail patients at risk for each outcome over time

The 2-year OS rate was 88% (CI 0.67–1), EFS was 79% (CI 0.56–1), and GRFS at 2 years was 70% (CI 0.47–1) (Fig. 5). Figure 1 demonstrates that most complications occurred within the first year post-transplant. Beyond this period, patients experienced no long-term complications requiring continuous disease-associated immunosuppression or hospital readmission. All IEI patients were successfully tapered off immunosuppression, and at the most recent follow-up, no patient had active GvHD.

## Discussion

Our study demonstrates that a comprehensive transplant approach—including myeloablative conditioning, *ex vivo* αβTCR/CD19-depleted allograft, personalized transplantation-associated immunosuppression and the ability to provide pre-emptive DLI—achieves promising outcomes in adults with IEI.

Although our study is limited by the low number of patients (*N* = 10) with a heterogeneous spectrum of IEI, this study offers valuable insights to tailor allo-HSCT for adults with IEI. First, we propose that in patients with IEI additional immunosuppression should be considered peri-transplant. In our approach, standard transplant-associated immunosuppression consists of MMF for 28 days [12]. In the first six patients all other immunosuppressive medication was stopped at the start of conditioning, except for the prednisolone in patient #6 which was slowly tapered to prevent secondary adrenal insufficiency. In patients #1 and #6, both diagnosed with CTLA4-HI, the post-transplantation period was marked by significant complications, including severe mucositis, non-infectious fever, multiple infections and GvHD, which led to prolonged hospitalization and death in one patient. The observed incidence of grade 3–4 aGvHD in two out of the first six patients was markedly higher to what is observed in adults with malignant diseases, who are transplanted with an αβTCR/CD19-depleted allograft, where a CI of aGvHD 3–4 of 0%-5% is reported [11, 12]. We therefore postulate that patients with IEI requiring high levels of disease-associated immunosuppression prior to allo-HSCT may need additional GvHD prophylaxis. In patients #7 and #9 prolonged disease-associated immunosuppression was administered and none of these patients developed severe GvHD (Fig. 1). Larger studies and subsequent identification of risk factors or biomarkers for increased risk of inflammation and GvHD are mandatory to provide guidelines on how peri transplant-associated immunosuppression can be personalized in this heterogeneous patient group [21].

Secondly, αβTCR/CD19 depletion may contribute to a favorable long-term toxicity profile. In allo-HSCT, extensive cGvHD is a major contributor to late treatment-related morbidity and mortality [22]. In our study, none of the patients developed extensive cGvHD. Another significant cause of late morbidity after allo-HSCT is infection [22]. Peri-transplantation viral reactivations are observed in up to 90% of SCT recipients [23]. Therefore, the observed rates of viral reactivation in this cohort are in line with what can be expected in this patient population. With letermovir prophylaxis (administered here only in patients 9 and 10), the incidence of CMV reactivation is expected to be reduced. Except for patient #6 with poorly controlled inflammation post-HSCT, no severe infections leading to hospitalization were observed beyond the first 3 months (Fig. 1, Online Resource Fig. S2). This might be attributed to the short duration of systemic immunosuppression (Fig. 1; Fig. 2), or that the immunoglobulin levels remained within the normal range (Fig. 3) in most patients.

Third, we aim to evaluate predictors for long-term success. Although clear criteria are lacking, reaching complete chimerism, sufficient response to vaccination and evaluation of IEI-related complications are considered important [22]. Mixed chimerism is associated with very late (> 5 years) complications after allo-HSCT [24]. Patients #5 and #6 showed mixed chimerism (Fig. 4). Both patients achieved full chimerism after pre-emptive DLI, without the development of GHVD, also when administered > 2 years after allo-HSCT. Post-HSCT vaccination is considered mandatory for all patients [16]. We found that that only one out of seven evaluable patients did not show seroprotection against *S. pneumoniae*, which is in line with what can be expected after allo-HSCT [25]. The patient without seroprotection was patient #8 with a late loss in chimerism, which might have contributed to this poor response. Re-vaccination was planned after successful DLI. Ultimately, allo-HSCT aims to resolve IEI-related symptoms and prevent the development of new symptoms. A recent retrospective analysis compared long term outcomes of adults with IEI and allo-HSCT to matched ‘non-transplanted’ controls based on patient and IEI characteristics. The disease-free survival at 5 years was superior in transplanted patients (58% vs 33%). In transplanted patients, most severe events and deaths occur within the first year after allo-HSCT, whereas in the non-transplant group, events keep accumulating over time, as expected [26]. Apart from severe and sometimes life-threatening events, having IEI implies lifelong follow-up, frequently chronic medication and/or immunoglobulin substitution and suffering from disease related symptoms. In our study, we used IDDA scores to evaluate the clinical burden of IEI and the impact of allo-HSCT. Our findings demonstrate that even in patients with a high disease burden, IDDA scores continue to improve beyond one year after allo-HSCT (Table 2, Online Resource Fig. S2). We propose that organ-specific symptom monitoring in transplanted IEI patients is a valuable tool for assessing and evaluating allo-HSCT outcomes.

In conclusion, our findings illustrate that allo-HSCT with αβTCR/CD19-depleted grafts is also feasible in adults with IEI. Engraftment rates and immune reconstitution appear encouraging, while the incidence of cGVHD is low, albeit these results require confirmation in larger, prospective studies. Our data contribute to the accumulating evidence on long term outcomes of stem cell transplantation [5, 26], which suggests that also for adult patients with severe IEI allo-HSCT should be considered as a treatment option.

## Supplementary Information

Below is the link to the electronic supplementary material.

Supplementary file1 (PDF 427 KB)

## Data Availability

No datasets were generated or analysed during the current study.
