# Supplementary material for: αβT/CD19-depleted Allogeneic Stem Cell Transplantation in Adults with Inborn Errors of Immunity
Source: J Clin Immunol. 2026 Feb 3;46(1):21. doi: 10.1007/s10875-025-01978-9 (PMC12913266; doi:10.1007/s10875-025-01978-9)
Supplement: Supplementary file 1 — Supplementary file1 (PDF 427 KB) [file 10875_2025_1978_MOESM1_ESM.pdf]

αβT/CD19-depleted allogeneic stem cell transplantation in adults with inborn errors of immunity

Journal of Clinical Immunology

Janneke J.H. de Winter*[1,2] MD, PhD, Birtan M. Ibrahimov*[1] BSc, Frances A. Verheij[1], Iris D. Brinkman[1], Anniek H.G. Stuut[3] MD, Pleun Schonewille[1] MSc, Marloes W. Heijstek[4] MD, PhD, Anna van Rhenen[1] MD, PhD, Lotte E. van der Wagen[1] MD, PhD, Laura G.M. Daenen[1] MD, PhD, Anke Janssen[1] MD, PhD, Tim J.A. Hutten[5] PhD, Jürgen Kuball[3] MD, PhD, Helen L. Leavis#[4] MD, PhD, Moniek A. de Witte#[1] MD, PhD

(1) Department of Hematology, University Medical Center Utrecht, Utrecht, Netherlands (2) Department of Hematology, Isala Clinics, Zwolle, Netherlands (3) Department of Hematology and Center of Translational Immunology, University Medical Center Utrecht, Utrecht, Netherlands (4) Department of Rheumatology and Clinical Immunology, University Medical Center Utrecht, Utrecht, Netherlands (5) Central Diagnostic Laboratory, University Medical Center Utrecht, Utrecht, Netherlands

**and # equal contributions*

Corresponding author

Moniek de Witte
Heidelberglaan 100, 3584 CX Utrecht, The Netherlands
Email: m.a.dewitte-7@umcutrecht.nl

Online Resource (Supplemental Data)

Table S1 Genetic variants of inborn errors of immunity

#	Diagnosis	Causal genetic variant
1	CTLA4HI	CTLA4 (NM_005214.4):c.567+5G>T p.(?)
2	APDS	PIK3R1 (NM_181523.2):c.1425+1G>A p.(?)
3	DOCK8	DOCK8 (NM_203447.4):c.743_1679+1del p.(Asp249fs) compound heterozygous with c.3389A>G p.(Gln1130Arg)
4	ALPS	FAS (NM_000043.30):c.809C>G (p.Thr270Arg)
5	DADA2	ADA2 (NM_001282225.1):c.506G>A p.(Arg169Gln) compound heterozygous with c.916C>T p.(Arg306*)
6	CTLA4HI	NC_000002.11:g.204731634_204983942del ~252kb deletion (CNV) including genes CTLA4 and ICOS
7	VEXAS syndrome	UBA1 (NM_003334.4):c.122T>C p.(Met41Thr)
8	CVID2 (TACI)	TNFRSF13B (NM_012452.2):c.517G>A p.(Ala173Thr)
9	IEI HA20	TNFAIP3 (NM_001270508.2):p.Tyr306*
10	DADA2	ADA2 (NM_001282225.1):p.(Arg169Gln)

ALPS: Autoimmune Lymphoproliferative Syndrome, APDS: Activated PI3K Delta Syndrome, CTLA4HI: Cytotoxic T-lymphocyte Associated Protein 4 Haploinsufficiency, CVID: Common Variable Immunodeficiency, DADA2: Adenosine Deaminase 2 Deficiency, DOCK 8: Dedicator of Cytokinesis 8 Deficiency, IDDA: Immune Deficiency and Dysregulation Activity, IEI: Inborn Errors of Immunity, TACI: Transmembrane Activator and CAML Interactor mutation, VEXAS syndrome: Vacuoles, E1 Enzyme, X-linked, Autoinflammatory, Somatic Syndrome

Table S2 Graft composition per patient

Graft Composition					
#	$CD34^+$/kg	αβ TCR cell/kg	γδ TCR cell/kg	$CD19^+$ B cells/kg	$CD16/56^+$ NK cells/kg
1	6.611	0.006	3.908	0.021	31.471
2	5.943	0.013	7.857	0.026	37.456
3	5.909	0.008	12.234	0.026	27.341
4	4.903	0.010	14.504	0.042	18.442
5	5.798	0.003	9.626	0.007	10.456
6	4.921	0.013	1.275	0.014	27.188
7	7.473	0.199	22.770	0.019	24.552
8	6.567	0.001	7.015	0.011	17.559
9	3.420	0.034	15.493	0.018	36.922
10	7.075	0.002	5.438	0.019	39.941
Median (range)	5.93 (3.42 – 7.47)	0.009 (0.001 – 0.199)	8.74 (1.27 – 22.8)	0.019 (0.007 – 0.042)	27.3 (10.5 – 39.9)

All doses are represented in 10^6 cells per kg. NK cells: natural killer cells, TCR: T cell receptor.

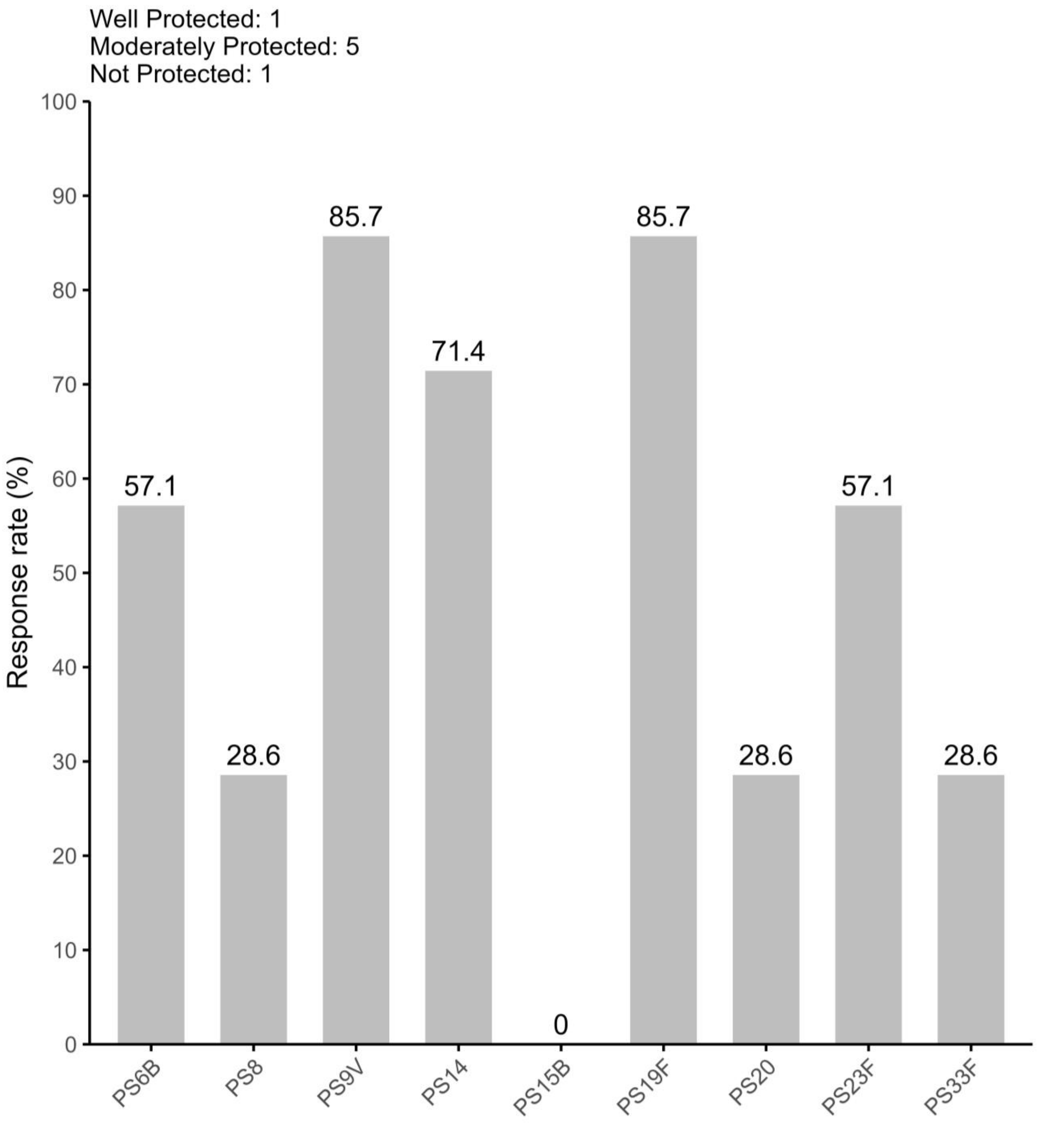

Fig. S1 Pneumococcal seroconversion by serotype. Figure illustrates the percentage of persons achieving protective antibody levels (≥0.35 µg/mL) against nine pneumococcal serotypes (PS6B, PS8, PS9V, PS14, PS15B, PS19F, PS20, PS23F, PS33F). Well protected: 1, moderately protected: 5, not protected: 1.

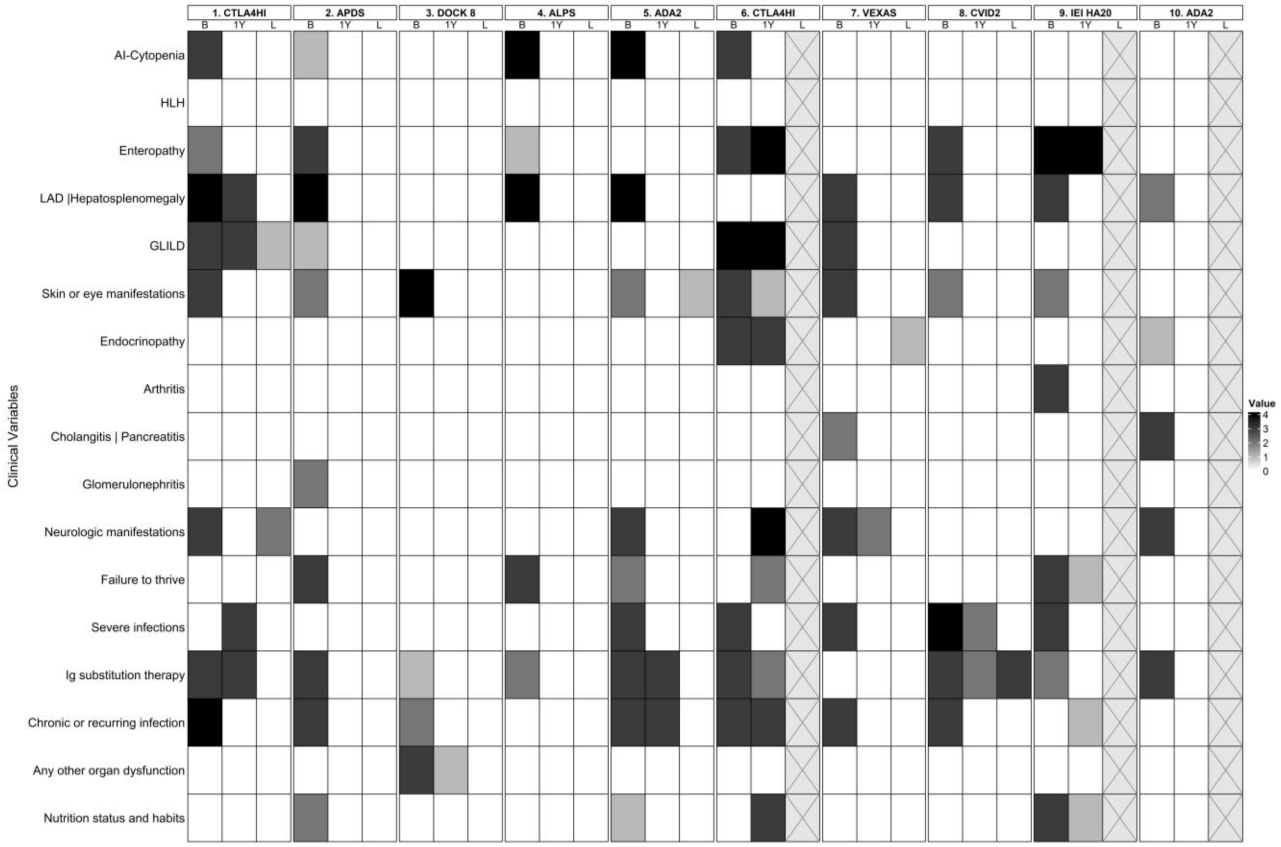

Fig. S2 Clinical manifestations and disease progression across time points in patients with immune dysregulation disorders. Figure illustrates clinical variables for 10 patients at baseline (B), 1 year (1Y), and the latest follow-up (L) after SCT. Darker shading represents severity (scale 0-4), corresponding with IDDA scoring. Gray crossed-out cells indicate missing data. AI: Autoimmune, GLILD: granulomatous lymphocytic interstitial lung disease, HLH hemophagocytic lymphohistiocytosis, Ig: immunoglobulin, LAD: lymphadenopathy.
